# An Unexpected Cardiac Arrest After Spinal Anesthesia for a Cesarean Section: A Case Report

**DOI:** 10.7759/cureus.92688

**Published:** 2025-09-19

**Authors:** Carolina Madruga, Maria Inês Taborda, Douglas Levier, Ana Faísco, Ângela Rodrigues

**Affiliations:** 1 Department of Anesthesiology, Unidade Local de Saúde de Amadora/Sintra, Amadora, PRT

**Keywords:** bezold-jarisch reflex, cesarean section, maternal cardiac arrest, neuraxial block, spinal anesthesia

## Abstract

Cardiac arrest during pregnancy is a rare but potentially catastrophic event, often requiring rapid, coordinated, multidisciplinary intervention. Neuraxial anesthesia, while generally considered safe and commonly used in obstetric settings, may be associated with severe cardiovascular complications. Among the proposed mechanisms, the Bezold-Jarisch reflex has been implicated in cases of sudden bradycardia and asystole following spinal anesthesia.

We report the case of a 39-week pregnant woman who was admitted for urgent cesarean delivery after failed induction of labor. The patient had a history of chronic hypertension, type 2 diabetes mellitus, and obesity, but was clinically stable at baseline. Spinal anesthesia was performed with co-loading of 500 mL of Ringer’s lactate. Shortly after the block and repositioning to the supine position, she developed marked hypotension (mean arterial pressure (MAP) < 60 mmHg), severe bradycardia, and subsequent asystole. Cardiopulmonary resuscitation was initiated immediately, with return of spontaneous circulation (ROSC) after administration of 1 mg of intravenous (IV) epinephrine and four minutes of effective chest compressions. An emergent cesarean section was performed under general anesthesia, with delivery of a viable neonate. Postoperative evaluation revealed no underlying cardiac or obstetric pathology, and the patient was discharged with full neurological recovery.

A comprehensive diagnostic approach was undertaken, including exclusion of maternal, medical, obstetric, and anesthetic-related causes of cardiac arrest. The temporal association between spinal anesthesia and cardiovascular collapse, in the absence of hemorrhage or underlying cardiac disease, supports the Bezold-Jarisch reflex as the most likely etiology. In this context, reduced preload - likely influenced by prolonged hospitalization and the relatively limited preloading volume of 500 mL - may have contributed to reflex activation in this patient.

This case underscores the importance of early recognition of vagally mediated reflexes, such as the Bezold-Jarisch reflex, and highlights the need for prompt resuscitation and coordinated multidisciplinary management. Adequate fluid resuscitation and heightened vigilance are essential to prevent catastrophic outcomes in obstetric anesthesia.

## Introduction

Neuraxial anesthesia is frequently the anesthetic technique of choice for elective and urgent cesarean deliveries due to its favorable safety profile in high-risk obstetric patients and decreased neonatal exposure to systemic drugs. Despite its general use, neuraxial anesthesia does not come without risks.

Cardiac arrest during delivery is a relatively rare event, with an incidence of approximately 1 in 12,000 to 1 in 36,000 deliveries [1-3]. It may occur unexpectedly, even in otherwise healthy pregnant women, posing risks of both maternal and fetal morbidity and mortality. The pregnant woman may be predisposed to complications during neuraxial blocks by the physiological changes of pregnancy, such as increased sensitivity to anesthetic drugs and altered cardiovascular dynamics.

Among the mechanisms involved in cardiac arrest after neuraxial anesthesia, the Bezold-Jarisch reflex - a cardioinhibitory response to reduced venous return and increased vagal tone - has been reported. It is characterized by paradoxical bradycardia, hypotension, and vasodilation after cardiac mechanoreceptor stimulation. This reflex may be triggered by sudden decreases in preload and sympathetic tone after spinal anesthesia [4,5].

In this scenario, the clinical team should rapidly consider a wide differential diagnosis of perioperative maternal cardiac arrest (MCA). This includes maternal, medical, obstetric, and anesthetic causes, with the aim of identifying reversible etiologies and guiding specific treatment. Even when a definitive diagnosis cannot be established, management should focus on high-quality resuscitation and multiorgan support therapy, with a systematic ABCDE (Airway, Breathing, Circulation, Disability, Exposure) approach. As per current international guidelines, perimortem cesarean section (PMCS) should be commenced within the first four to five minutes of MCA to improve both maternal and fetal outcomes [6].

In this report, we present the case of a 39-week pregnant female who experienced a sudden cardiac arrest soon after spinal anesthesia for an urgent cesarean section. Early recognition and effective response by the anesthetic and surgical teams played a critical role in the patient's full recovery. This case highlights the need for heightened vigilance regarding vagally mediated reflexes, such as the Bezold-Jarisch reflex, as well as the critical role of prompt resuscitation and multidisciplinary coordination in the obstetric operating room.

## Case presentation

A 30-year-old, 39-week pregnant woman (gravida 2, para 1) was admitted for urgent cesarean section due to arrested labor. Prior to this decision, she had been undergoing labor induction for three days with dinoprostone (prostaglandin E2), without achieving active labor progression. Her medical history included chronic hypertension, type 2 diabetes mellitus, and class II obesity (BMI 38 kg/m²). She was being treated with methyldopa for hypertension and a combination of metformin and insulin for glycemic control. At the time of preoperative evaluation, the patient was clinically stable and euglycemic, with no known drug allergies or history of cardiovascular or neurological disease. Baseline vital signs - including heart rate, blood pressure, and oxygen saturation - were within normal limits.

Spinal anesthesia was performed in the sitting position at the L3-L4 interspace using 8.5 mg of hyperbaric bupivacaine 0.5%, 2.5 mcg of sufentanil, and 50 mcg of preservative-free morphine. At the time of the block, the patient was receiving 500 mL of Ringer’s lactate. After injection and repositioning to the supine position with left uterine displacement, the only drug administered was prophylactic cefazolin, 2 g intravenously.

Within minutes, the patient developed severe hypotension (mean arterial pressure (MAP) < 60 mmHg), refractory to 10 mg of intravenous (IV) ephedrine, rapidly progressing to unresponsiveness, bradycardia, and asystole. Cardiopulmonary resuscitation was immediately initiated, with return of spontaneous circulation (ROSC) after 1 mg of epinephrine and approximately four minutes of chest compressions combined with manual left uterine displacement. The patient was intubated, ventilated, and started on norepinephrine infusion to maintain hemodynamic stability. Oxygenation was preserved, with SpO₂ consistently above 95% on FiO₂ 30%. Importantly, no cutaneous manifestations, bronchospasm, or distributive shock features were observed, which argued strongly against anaphylaxis as a contributing factor.

An emergent cesarean section was performed under general anesthesia. The uterus and adnexa appeared normal, with no signs of rupture, abruption, or placenta accreta. Estimated blood loss was 700 mL. Intraoperatively, the patient received approximately 1500 mL of IV crystalloids, with no need for blood product administration.

Following resuscitation, an arterial line was placed for invasive hemodynamic monitoring, and laboratory investigations revealed elevated troponin T (TnT 236 ng/L) and creatine phosphokinase-MB (CK-MB 213 U/L), consistent with myocardial injury. These findings were interpreted as most likely reflecting post-arrest myocardial stunning rather than a primary type 2 myocardial infarction. Other laboratory parameters, including coagulation, complete blood count, renal and hepatic function, and electrolytes, were within normal limits (Table 1). Arterial blood gas analysis showed metabolic acidosis with mild hyperlactatemia (pH 7.24, lactate 3 mmol/L).

**Table 1:** Laboratory findings following cardiac arrest NT-proBNP, N-terminal pro-B-type natriuretic peptide; MCA, maternal cardiac arrest

Laboratory findings	(Reference range) units	Values	
		Baseline	After MCA
Complete Blood Count			
Hemoglobin	(12.0 - 15.0) g/dL	11.8	11.5
Erythrocytes	(3.80 - 4.80) x 10^12^/L	4.40	4.29
Hematocrit	(36.0 - 46.0) %	37	36
Leukocytes	(4.0 - 10.0) x 10^9^/L	14.0	21.7
Neutrophiles	(2.0 - 7.0) x 10^9^/L	11.9	19.6
Eosinophiles	(0.0 - 0.5) x 10^9^/L	0.0	0.0
Basophiles	(0.0 - 0.1) x 10^9^/L	0.0	0.0
Lymphocytes	(1.0 - 3.0) x 10^9^/L	1.1	1.0
Monocytes	(0.2 - 1.0) x 10^9^/L	1.0	1.1
Platelets	(150 - 410) x 10^9^/L	267	251
Coagulation			
Prothrombin Time	(9.7 - 11.8) sec	10.3	10.5
INR	(< 1.2)	0.9	1.0
Activated Partial Thromboplastin Time	(20.6 - 29.5) sec	30.0	32.0
Fibrinogen	(1.8 - 3.5) g/L	-	6.0
D-dimer	(< 500) µg/L	-	11968
Serum Biochemistry			
Creatinine	(0.5 - 0.9) mg/dL	0.65	0.74
Urea	(< 50) mg/dL	15	23.1
Sodium	(136 - 145) mmol/L	138	136.4
Potassium	(3.5 - 5.1) mmol/L	4.2	4.05
Chloride	(98 - 107) mmol/L	105	103.6
Aspartate Aminotransferase (AST)	(< 32) U/L	19	19
Alanine Aminotransferase (ALT)	(< 33) U/L	14	15
Alkaline Phosphatase (ALP)	(35 - 105) U/L	120	128
Gamma-Glutamyl Transferase (GGT)	(< 40) UI/L	16	16
Total Bilirubin	(≤ 1.2) mg/dL	0.78	0.98
Lactate Dehydrogenase (LDH)	(135 - 214) U/L	-	208
Total Creatine Kinase (Total CK)	(26 - 192) U/L	-	233.76
Glucose	(74 - 109) mg/dL	140	205
Cardiac Biomarkers			
Creatine Phosphokinase-MB (CK-MB)	(< 25) U/L	-	213
Troponin T (TnT)	(< 0.05) ng/mL	-	236
NT-proBNP	(< 125) pg/mL	-	50.7
Arterial Blood Gas (ABG)			
pH	(7.37 - 7.45)	-	7.24
pCO_2_	(35 - 46) mmHg	-	39.9
pO_2_	(70 - 100) mmHg	-	164
Oxygen Saturation	(> 96) %	-	99
Bicarbonate (HCO_3_^-^)	(21 - 26) mmol/L	-	16.8
Anion Gap	(8 - 16) mmol/L	-	15
Lactate	(< 1.8) mmol/L	-	3

The patient delivered a live neonate with 6/8/8 Apgar scores, who was transferred to the neonatal intensive care unit (ICU).

**Figure 1:**
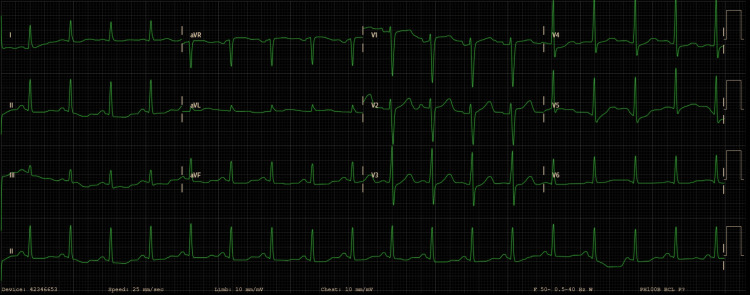
A 12-lead electrocardiogram Postoperative 12-lead electrocardiogram showing sinus tachycardia without ischemic changes.

Following the surgical procedure, the patient was transferred to the ICU for post-arrest care and to investigate the etiology of the MCA. A 12-lead electrocardiogram (ECG), performed in the immediate postoperative period, revealed sinus tachycardia with no signs of acute ischemia (Figure 1). Transthoracic echocardiography, performed by the consultant cardiologist, showed a mildly depressed global left ventricular systolic function (estimated ejection fraction of 45%), without segmental wall motion abnormalities, significant valvular disease, or pericardial effusion (Videos 1-3).

**Video 1:** Transthoracic echocardiogram: parasternal long-axis (PLAX) view PLAX echocardiographic view demonstrating no segmental wall motion abnormalities or right ventricular dilation.

**Video 2:** Transthoracic echocardiogram: apical four-chamber (A4C) view A4C echocardiographic view illustrating reduced global left ventricular contractility with preserved right ventricular size and function.

**Video 3:** Transthoracic echocardiogram: parasternal short-axis (PSAX) view, level of papillary muscles PSAX echocardiographic view showing diffuse hypokinesia of the left ventricle, consistent with global systolic dysfunction, and no evidence of right ventricular overload.

In the ICU, the patient was extubated five hours after admission, and a subsequent neurological examination confirmed complete recovery. She was discharged home on the fifth day postpartum, with no further medical or surgical complications.

The elevation of cardiac biomarkers, consistent with myocardial injury, together with the echocardiographic findings of diffuse hypokinesia, was interpreted as secondary to the cardiac arrest. Serial measurements showed a sustained decline (Table 2), further supporting this interpretation. Nevertheless, the patient was referred for ongoing cardiology follow-up to ensure complete recovery and to exclude underlying cardiac pathology. An echocardiogram performed six months after discharge demonstrated full recovery of global left ventricular systolic function, with an estimated ejection fraction of 58%.

**Table 2:** Evolution of the cardiac biomarkers over time NT-proBNP, N-terminal pro-B-type natriuretic peptide; MCA, maternal cardiac arrest

Laboratory findings	(Reference range) units	Values			
		After MCA	12h later	36h later	6 months later
Cardiac Biomarkers					
Total Creatine Kinase (Total CK)	(26 - 192) U/L	233.76	-	-	72
Creatine Phosphokinase-MB (CK-MB)	(< 25) U/L	213	-	33	-
Troponin T (TnT)	(< 0.05) ng/mL	236	141	60	-
NT-proBNP	(< 125) pg/mL	50.7	1630	-	13

## Discussion

This case illustrates a comprehensive approach to MCA following spinal anesthesia for cesarean section. Although rare, cardiac arrest in pregnant women is a potentially catastrophic event, further complicated by the physiological interplay between two patients - mother and fetus. A multidisciplinary team must be prepared to provide high-quality resuscitation while accounting for the physiological changes of pregnancy and promptly identifying potential underlying causes.

The etiology can be broadly categorized into three major groups: maternal medical conditions, obstetric complications, and anesthesia-related factors.

Maternal medical causes

Pre-existing comorbidities, such as congenital or acquired heart disease, cardiomyopathies, arrhythmias, thromboembolic events, and metabolic disturbances, may increase the risk of cardiac decompensation during the physiological stress of labor and surgery [6].

In our case, despite the presence of chronic hypertension, diabetes, and obesity - recognized risk factors for peripartum complications - there was no evidence of underlying structural or functional heart disease, nor of arrhythmias. The postoperative ECG showed sinus tachycardia without ischemia-related changes, and echocardiography demonstrated globally depressed but non-segmental systolic function, consistent with myocardial stunning secondary to cardiac arrest rather than a primary pathology. Laboratory evaluation revealed a transient elevation of cardiac biomarkers, supporting myocardial injury, although serial measurements showed a sustained decline consistent with secondary damage from the arrest. Other laboratory results were unremarkable, including normal electrolyte balance, glycemia, and coagulation parameters, consistent with the absence of major bleeding complications during the cesarean section.

Thromboembolic events, including pulmonary embolism (PE), represent a major non-obstetric cause of maternal mortality and should be considered in all cases of sudden cardiovascular collapse [7]. However, in this patient, there were no signs of hypoxemia or right ventricular strain on echocardiography, nor clinical findings suggestive of deep vein thrombosis or respiratory compromise. The absence of electrocardiographic changes and the rapid ROSC further reduce the likelihood of PE as a contributing factor. Collectively, these findings suggest that maternal medical conditions were unlikely contributors.

Obstetric causes

Obstetric emergencies, such as amniotic fluid embolism (AFE), uterine rupture, placental abruption, and massive hemorrhage, are important etiologies of MCA [6].

AFE can present abruptly with hypoxia, hypotension, and cardiovascular collapse. It is hypothesized to result from a maternal anaphylactoid reaction to fetal material entering the pulmonary circulation, leading to sudden apnea, bradycardia, and hypotension. The diagnosis is notoriously challenging and often remains clinical, with definitive confirmation typically only possible at autopsy [8,9]. However, the absence of early noticeable hypoxia prior to the arrest, the lack of coagulopathy, and the rapid hemodynamic recovery within four minutes make this an unlikely diagnosis in this patient. Patients with true AFE typically require prolonged mechanical ventilation and extended ICU support, which was not necessary in this case.

Hypertensive disorders of pregnancy, including preeclampsia, eclampsia, and HELLP syndrome, are also important considerations due to their potential to cause severe endothelial dysfunction, coagulopathy, intracranial hemorrhage, and multiorgan failure [6]. Although this patient had chronic hypertension, there were no clinical or laboratory findings suggestive of superimposed preeclampsia, nor evidence of neurological symptoms, hemolysis, elevated liver enzymes, or thrombocytopenia to support a diagnosis of HELLP syndrome.

Furthermore, in this case, there was no clinical or intraoperative evidence of embolic phenomena, coagulopathy, or abnormal bleeding. Uterine tone was adequate, estimated blood loss was within expected limits, and the cesarean section proceeded without obstetric complications. There were also no signs of uteroplacental disruption, making obstetric causes of cardiac arrest unlikely contributors in this context.

Anesthetic-related causes

Anesthetic complications remain an important cause of perioperative cardiac arrest in parturients, especially with neuraxial anesthesia. Potential mechanisms include high or total spinal block, unopposed vagal tone due to sympathetic blockade at the level of the cardiac accelerator fibers (T1-T4), activation of the Bezold-Jarisch reflex, systemic toxicity of local anesthetics, and anaphylaxis [6,10]. Among these, the first three are most commonly implicated in sudden bradycardia and asystole following spinal anesthesia.

In this case, the close temporal relationship between the spinal block and the cardiovascular collapse strongly supports an anesthetic mechanism. The procedure was performed with standard doses of hyperbaric bupivacaine and opioid adjuvants, and the patient was appropriately repositioned with a slight left lateral tilt. However, within minutes, she developed hypotension unresponsive to ephedrine, followed by unresponsiveness, severe bradycardia, and asystole.

Although high or total spinal block is a well-known complication of neuraxial anesthesia, the absence of signs such as upper limb weakness or apnea prior to the arrest makes this diagnosis less likely. Because of the rapid onset of cardiovascular collapse, sensory level assessment (to pinprick or cold) could not be performed before the event, which limited immediate confirmation of block height. Opioid-induced respiratory depression is a complication of intrathecal opioid administration, secondary to its cephalad spread in cerebrospinal fluid. In this case, the abrupt onset of bradycardia and asystole within minutes of spinal anesthesia, in the absence of preceding hypoventilation or hypoxia, suggests this etiology is unlikely. Moreover, the patient required no prolonged ventilatory support, further arguing against opioid-related respiratory compromise.

Instead, the clinical course is highly suggestive of the Bezold-Jarisch reflex - a cardioinhibitory response triggered by decreased venous return and enhanced vagal activity. In obstetric patients, this reflex may be potentiated by aortocaval compression, rapid sympathetic blockade, and relative hypovolemia.

In this context, inadequate intravascular volume loading may have contributed. The patient had been admitted for a prolonged labor induction over three days. Insufficient volume resuscitation prior to spinal anesthesia may have exacerbated the drop in preload at the time of sympathetic block. Since adequate preload is essential to counteract this risk, the combination of reduced venous return and heightened vagal tone could have precipitated activation of the Bezold-Jarisch reflex. Preventive strategies, such as preloading or co-loading with crystalloids or colloids, together with the early initiation of vasopressors in high-risk parturients, are therefore recommended to reduce the likelihood of this complication.

Importantly, there was no evidence of local anesthetic systemic toxicity or allergic reaction, and the anesthetic technique and drug doses were appropriate. The absence of hemorrhagic complications, in combination with normal postoperative laboratory values and preserved neurologic function, further supports a neurally mediated reflex as the most likely etiology.

## Conclusions

MAC in the setting of spinal anesthesia remains a rare but life-threatening event. A systematic and time-sensitive diagnostic approach is essential to guide immediate resuscitation and identify reversible causes. In the present case, thorough multidisciplinary evaluation and a favorable clinical course suggest that major obstetric, metabolic, and cardiac causes were unlikely. The combination of reduced preload, rapid sympathetic blockade, and increased vagal tone is consistent with the Bezold-Jarisch reflex as the most plausible mechanism for the cardiovascular collapse.

This case underscores that even clinically stable parturients may experience unexpected cardiac arrest following spinal anesthesia, highlighting the importance of adequate fluid resuscitation, early vasopressor support, and vigilance for neurally mediated reflexes. Prompt recognition, adherence to obstetric resuscitation protocols, and timely perimortem cesarean delivery, when indicated, are critical to optimizing maternal and fetal outcomes. Multidisciplinary preparedness remains a cornerstone of safe obstetric anesthesia.

## References

[1] Mhyre JM, Tsen LC, Einav S, Kuklina EV, Leffert LR, Bateman BT (2014). Cardiac arrest during hospitalization for delivery in the United States, 1998-2011. Anesthesiology.

[2] Balki M, Liu S, León JA, Baghirzada L (2017). Epidemiology of cardiac arrest during hospitalization for delivery in Canada: a nationwide study. Anesth Analg.

[3] Beckett VA, Knight M, Sharpe P (2017). The CAPS study: incidence, management and outcomes of cardiac arrest in pregnancy in the UK: a prospective, descriptive study. BJOG.

[4] Mackey DC, Carpenter RL, Thompson GE, Brown DL, Bodily MN (1989). Bradycardia and asystole during spinal anesthesia: a report of three cases without morbidity. Anesthesiology.

[5] Kinsella SM, Tuckey JP (2001). Perioperative bradycardia and asystole: relationship to vasovagal syncope and the Bezold-Jarisch reflex. Br J Anaesth.

[6] Jeejeebhoy FM, Zelop CM, Lipman S (2015). Cardiac arrest in pregnancy: a scientific statement from the American Heart Association. Circulation.

[7] Cantwell R, Clutton-Brock T, Cooper G (2011). Saving mothers' lives: reviewing maternal deaths to make motherhood safer: 2006-2008. The eighth report of the confidential enquiries into maternal deaths in the United Kingdom. BJOG.

[8] Clark SL, Hankins GD, Dudley DA, Dildy GA, Porter TF (1995). Amniotic fluid embolism: analysis of the national registry. Am J Obstet Gynecol.

[9] Kaur K, Bhardwaj M, Kumar P, Singhal S, Singh T, Hooda S (2016). Amniotic fluid embolism. J Anaesthesiol Clin Pharmacol.

[10] Pollard JB (2001). Cardiac arrest during spinal anesthesia: common mechanisms and strategies for prevention. Anesth Analg.

